# The association between obesity, diet quality and hearing loss in older adults

**DOI:** 10.18632/aging.101717

**Published:** 2019-01-04

**Authors:** Pauline H. Croll, Trudy Voortman, Meike W. Vernooij, Robert J. Baatenburg de Jong, Frank R. Lin, Fernando Rivadeneira, M. Arfan Ikram, André Goedegebure

**Affiliations:** 1 Department of Otorhinolaryngology, Head and Neck Surgery, Erasmus University Medical Center, Rotterdam, the Netherlands; 2 Department of Epidemiology, Erasmus University Medical Center, Rotterdam, the Netherlands; 3 Department of Radiology and Nuclear Medicine, Erasmus University Medical Center, Rotterdam, the Netherlands; 4 Department of Otolaryngology-Head & Neck Surgery and Epidemiology, Johns Hopkins School of Medicine and Bloomberg School of Public Health, Baltimore, MD 21205, USA; 5 Department of Internal Medicine, Erasmus University Medical Center, Rotterdam, the Netherlands

**Keywords:** age-related hearing loss, aging, body composition, diet quality, fat mass index, fat-free mass index

## Abstract

Background: With the aging population, the prevalence of age-related hearing loss will increase substantially. Prevention requires more knowledge on modifiable risk factors. Obesity and diet quality have been suggested to play a role in the etiology of age-related hearing loss. We aimed to investigate independent associations of body composition and diet quality with age-related hearing loss.

Methods: We performed cross-sectional and longitudinal analyses (follow-up: 4.4 years) in the population-based Rotterdam Study. At baseline (2006-2014), 2,906 participants underwent assessment of body composition, diet, and hearing. Of these 2,906 participants, 636 had hearing assessment at follow-up (2014-2016). Association of body composition and of diet quality with hearing loss were examined using multivariable linear regression models.

Results: Cross-sectionally, higher body mass index and fat mass index were associated with increased hearing thresholds. These associations did not remain statistically significant at follow-up. We found no associations between overall diet quality and hearing thresholds.

Conclusions: This study shows that a higher body mass index, and in particular a higher fat mass index, is related to age-related hearing loss. However, whether maintaining a healthy body composition may actually reduce the effects of age-related hearing loss in the aging population requires further longitudinal population-based research.

## Introduction

Age-related hearing loss is highly prevalent among older adults [1,2], and is characterized by reduced hearing sensitivity and speech understanding, resulting from degeneration of the cochlea or the auditory nerves or both [3]. Hearing loss contributes to social isolation, depression, and possibly dementia [2,4–7]. With a growing and aging population, the number of people with hearing loss and its consequences will increase [1]. Therefore, prevention of hearing loss is key. But this requires more knowledge about modifiable risk factors.

One such risk factor may be obesity, which has been linked to increased hearing thresholds [4,5,8–10]. An important limitation is that previous studies mostly used body mass index (BMI) as a measure of body composition. Aging is associated with a decrease in lean mass and an increase in fat mass, making BMI less suitable as an approximation of body composition in the elderly [11]. Moreover, cross-sectional studies are inconclusive about the true association between obesity and hearing and therefore more longitudinal research is needed.

It has been suggested that diet quality plays a role in the relation between body composition and age-related hearing loss. Studies observed a relation between food groups, such as fish and carbohydrates consumption, and hearing thresholds [12–14]. However, many complex interactions occur across different food components and nutrients [15] which triggered the interest to study effects of dietary patterns as a whole [15]. Two other studies examined the relation between diet quality and age-related hearing loss, and in both found an association between better diet quality and poorer hearing [13,16]. But more research is needed to verify this association.

We aimed to investigate the association between detailed body composition (distinguishing between lean mass and fat mass) and age-related hearing loss and between overall diet quality and age-related hearing loss. We mutually adjusted for body composition and diet quality, therefore examining the independent relationship between body composition, diet quality and age-related hearing loss.

## RESULTS

Table 1 shows the population characteristics. Mean age at baseline was 66.1 years (standard deviation (SD): 7.33), and 56.7% of the participants were women. Participants had a mean BMI of 27.3 kg/m^2^ (SD: 4.1) and a median dietary adherence score of 7 (interquartile range: 6-8). Participants had a mean overall frequency hearing threshold of 24.1 decibel (dB) (SD: 12.1). Of the total group of 2,906 participants, 636 participants had a second hearing assessment at follow-up (median 4.4 years later, range 5.1) (Table 1).

**Table 1:** Population characteristics.

Characteristics	Baseline	Follow-up
	**N = 2,906**	**N = 636**
Age, years	**66.1 (7.3)**	
Female, %	**56.7**	
Education level, %		
Primary	**7.3**	
Lower	**36.6**	
Middle	**29.4**	
High	**25.9**	
Hypertension, %	**47.5**	
Hypercholesterolemia, %	**55.5**	
Diabetes, %	**8.2**	
Smoking, %		
Never	**32.1**	
Former	**52.4**	
Current	**15.0**	
Physical activity, MET-hours per week	**48.0 (IQR: 19.7-86.6)**	
		
*Baseline hearing thresholds*		
Overall frequency hearing loss, dB	**24.1 (12.1)**	
Low frequency hearing loss, dB	**14.5 (9.3)**	
High frequency hearing loss, dB	**32.6 (17.4)**	
		
*Hearing thresholds for participants with 2 hearing assessments (N = 636)*		
Overall frequency hearing loss, dB	**30.0 (10.8)**	**32.5 (11.3)**
Low frequency hearing loss, dB	**17.6 (9.1)**	**18.1 (10.0)**
High frequency hearing loss, dB	**41.4 (15.7)**	**46.2 (15.9)**
		
*Body composition*		
Length, cm	**170.0 (9.3)**	
Weight, kg	**79.1 (14.4)**	
Body mass index, kg/m^2^	**27.3 (4.1)**	
Fat mass index, kg/m^2^	**9.9 (3.2)**	
Fat free mass index, kg/m^2^	**17.4 (2.1)**	
		
*Dietary characteristics*		
Energy intake*	**2,119 (1,706-2,600)**	
Dietary guideline adherence score*	**7 (6-8)**	

Values are based on imputed data. Numbers of missings per variable were 360 for formerly smoking, 314 for never smoking and 207 for physical activity. Values are mean (standard deviation) for continuous variables or median (interquartile range) when indicated (*), percentages for dichotomous variables. MET: metabolic equivalent of task. dB: decibel. cm: centimeter. kg: kilogram. m: meter. IQR: interquartile range.

### Cross-sectional results

After adjustment for relevant confounders (model 2), one point higher BMI was associated with a 0.53 dB (CI: 0.04, 1.01) increase in hearing thresholds across all frequencies and with 0.42 dB (CI: 0.01, 0.82) increase in hearing thresholds in the lower frequencies (Table 2). Associations of BMI with hearing thresholds were mainly explained by fat mass index (FMI) rather than fat-free mass index (FFMI). One SD higher FMI was related to 0.58 dB (CI: 0.06, 1.09) increased hearing thresholds in all frequencies and with 0.43 dB (CI: -0.00, 0.86) increase in hearing thresholds among the lower frequencies (borderline non-significant: p = 0.05) (Table 2). When additionally adjusting for diet quality (model 3) effect estimates remained similar (Table 2). We did not find any associations between diet quality and hearing thresholds (Table 3, S2, and S3). However, consumption of unsaturated fats and oils was associated with increased hearing thresholds and consumption of sugar containing beverages was associated with decreased hearing thresholds (Table 3). Effects estimates for most associations remained similar between model 1 and model 2 (Table 2, 3, S2, and S3).

**Table 2:** The cross-sectional association between body composition and hearing thresholds.

	All frequencies	Low frequencies	High frequencies
	Difference in dB (CI 95%)	Difference in dB (CI 95%)	Difference in dB (CI 95%)
		**Model 1**	
Body mass index (SD)	**0.45 (0.09, 0.81)**	**0.35 (0.05, 0.65)**	**0.53 (0.01, 1.04)**
Fat mass index (SD)	**0.43 (0.05, 0.82)**	**0.37 (0.05, 0.70)**	0.47 (-0.08, 1.02)
Fat free mass index (SD)	**0.50 (0.05, 0.94)**	0.30 (-0.08, 0.67)	0.64 (0.00, 1.28)
		**Model 2**	
Body mass index (SD)	0.53 (0.04, 1.01)	0.42 (0.01, 0.82)	0.60 (-0.10, 1.30)
Fat mass index (SD)	0.58 (0.06, 1.09)	0.43 (-0.00, 0.86)	0.71 (-0.04, 1.46)
Fat free mass index (SD)	0.39 (-0.18, 0.96)	0.36 (-0.12, 0.83)	0.35 (-0.48, 1.17)
		**Model 3**	
Body mass index (SD)	0.52 (0.03, 1.00)	0.397 (-0.01, 0.80)	0.59 (-0.11, 1.29)
Fat mass index (SD)	0.56 (0.05, 1.08)	0.41 (-0.03, 0.84)	0.70 (-0.05, 1.45)
Fat free mass index (SD)	0.39 (-0.18, 0.96)	0.35 (-0.12, 0.83)	0.34 (-0.48, 1.17)

All frequencies (0.25, 0.50, 1, 2, 4, and 8 kHz); low frequencies (0.25, 0.50, and 1 kHz); high frequencies (2, 4, and 8 kHz). Difference represents the difference in dB per one SD higher body mass index, fat mass index, and fat free mass index. CI: confidence interval. Model 1: adjusted for sex, age, age^2^, and education. Model 2: additionally adjusted for energy intake, total brain volume, physical activity, smoking, alcohol, hypertension, hypercholesterolemia and type 2 diabetes. Model 3: additionally adjusted for diet quality score.

**Table 3:** The cross-sectional association between diet quality, food groups and hearing thresholds - model 3.

	All frequencies	Low frequencies	High frequencies
	Difference (CI 95%)	Difference (CI 95%)	Difference (CI 95%)
Diet quality	-0.09 (-0.34, 0.15)	-0.16 (-0.36, 0.05)	-0.05 (-0.40, 0.31)
Vegetables	-0.01 (-0.04, 0.01)	-0.02 (-0.04, 0.01)	-0.01 (-0.05, 0.03)
Fruit	-0.01 (-0.02, 0.01)	-0.00 (-0.02, 0.01)	-0.01 (-0.03, 0.01)
Whole grain products	-0.00 (-0.06, 0.06)	-0.03 (-0.08, 0.02)	0.03 (-0.06, 0.12)
Whole grains/total grains ratio	0.00 (-0.05, 0.06)	0.00 (-0.05, 0.05)	0.02 (-0.07, 0.10)
Legumes	6.93 (-2.72, 16.58)	8.04 (-0.00, 16.07)	4.51 (-9.46, 18.49)
Nuts	-0.01 (-0.31, 0.28)	-0.02 (-0.25, 0.22)	0.01 (-0.20, 0.22)
Dairy	0.00 (-0.01, 0.02)	-0.01 (-0.02, 0.01)	0.01 (-0.01, 0.04)
Fish	-0.06 (-0.26, 0.14)	-0.10 (-0.26, 0.07)	-0.06 (-0.35, 0.23)
Tea	-0.01 (-0.03, 0.01)	-0.01 (-0.02, 0.01)	-0.02 (-0.05, 0.01)
Unsaturated fats/total fats ratio	0.13 (-0.02, 0.29)	**0.16 (0.03, 0.29)**	0.13 (-0.09, 0.36)
Salt	0.00 (-0.00, 0.00)	-0.00 (-0.01, 0.00)	0.00 (-0.01, 0.01)
Alcohol	-0.07 (-0.36, 0.22)	-0.06 (-0.30, 0.19)	-0.09 (-0.52, 0.33)
Red and processed meat	0.04 (-0.06, 0.12)	0.05 (-0.02, 0.13)	0.01 (-0.13, 0.15)
Sugar containing beverages	-0.02 (-0.06, 0.01)	**-0.04 (-0.07, -0.01)**	-0.04 (-0.08, 0.00)

All frequencies (0.25, 0.50, 1, 2, 4, and 8 kHz); low frequencies (0.25, 0.50, and 1 kHz); high frequencies (2, 4, and 8 kHz). Difference represents difference in dB per 1 point increase in diet quality score on a scale ranging from 0 to 14 or a 10 gram increase for the individual food components. CI: confidence interval. Adjusted for sex, age, age^2^, education, physical activity, smoking (former and current), alcohol intake, hypertension, hypercholesterolemia, prevalent diabetes mellitus, total brain volume, energy intake and BMI (model 3). We did not adjust for alcohol intake in grams in the assessment of alcohol with hearing thresholds. Significant effect estimates (p<0.05) are indicated in **bold**.

### Longitudinal results

Body composition and diet quality were not related to change in hearing thresholds at follow-up (Table S1, S4, S5, and S6). Some food groups did show a significant relationship with hearing thresholds over time. For all frequencies, we found that higher intake of nuts was associated with a 0.95 (CI: -1.52, -0.37) dB decrease of hearing thresholds, as well as in the higher frequencies where higher intake of nuts was associated with a 1.10 dB (-1.87, -0.33) decrease of hearing thresholds (Table S6). Moreover, for all frequencies we found that higher vegetable intake was associated with a 0.05 dB (CI: -0.09, -0.00) decrease in hearing thresholds (Table S6). Effects estimates for most associations remained similar between model 1 and model 2 (Table S1, S4, S5, and S6)

There were no significant interactions (p<0.05) between body composition and sex, diet quality and BMI, and between diet quality and sex. Effect sizes did not differ between men and women (data not shown).

## DISCUSSION

In this large sample of community-dwelling individuals, we found that adiposity was associated with increased hearing threshold. Although not statistically significant, these effects estimates were similar at follow-up. We found no associations of overall diet quality with age-related hearing loss.

Strengths of our study included the population-based setting, the large sample size, and the standardized assessment of hearing thresholds with pure-tone audiograms and detailed measurement of body composition. Some limitations of this study should also be acknowledged. First, although we adjusted for possible confounders, there still might be residual confounding present. At last, the FFQ relies on an individual’s capacity to recall their dietary behaviour over the past month. Recall bias in dietary behaviour could be a systematic bias.

We found that a higher BMI was associated with higher hearing thresholds in our cross-sectional analysis. BMI is an important marker for metabolic diseases [17], and is a classic indicator for obesity. Other studies also confirmed this positive relationship between BMI and hearing thresholds [5,7–10,18,19]. However, some studies found non-significant associations [20–22], and therefore the true relationship between BMI and hearing thresholds in the elderly remains controversial. A new aspect of our study is that we differentiated between FMI and FFMI, which is thought to be a more accurate reflection of metabolic unhealthy people and metabolic healthy people [23]. As we found a significant effect for FMI and not for FFMI, it is possible that the absence of an association in other studies is explained by a more prominent contribution of FFMI to the BMI compared to FMI.

The fact that we find an association for FMI and not for FFMI is in line with the common idea that body composition influences hearing thresholds through vascular mechanisms. Hearing thresholds are associated with vasculopathies in metabolic diseases and therefore it is hypothesized that BMI is associated with the development of increased hearing thresholds [8,18,23]. The integrity of an individual’s auditory hair cells is paramount to their ability to detect sound [4], and a healthy blood flow and oxygen contribute to the health of these cells. As such, the underlying mechanism between age-related hearing loss and obesity may be due to the mechanical strain on the capillary walls caused by adipose tissue [4,23]. An animal study found narrowed blood vessels in the stria vascularis in mice with obesity [24]. This is a heavily vascularized part of the cochlea, therefore highly sensitive to any cardiovascular changes [2,4,25]. A similar vascular mechanism might be active in human older adults.

With the growing prevalence of obesity [4,26], healthy diet may serve as a modifiable risk factor for both hearing loss and obesity. Two other studies examined the effects of dietary patterns on hearing thresholds. Contrary to what we have found, they both report significant associations between diet quality and hearing thresholds [13,16], although the found association did not persist at follow-up [16]. Both studies did not adjust thoroughly for confounding, therefore residual confounding may be present in their results. Interestingly, the study of Spankovich et al. [13] compromises a broader age range, and found that diet quality was associated with hearing loss in their younger population. Similar to us, in their older population there was an association between body composition and hearing thresholds. As such, it might be that diet quality has a bigger effect in a younger population. More research has been conducted concerning food nutrients and age-related hearing loss, and those studies reported that sufficient intake of fish and whole grains, and moderate intake of alcohol are related to lower hearing thresholds [12,27–29], whereas we found a positive association between intake of unsaturated fats and oils and hearing thresholds and a negative association between intake of sugar containing beverages and hearing thresholds. Moreover, on follow-up we found that more consumption of vegetables and nuts associated with lower hearing thresholds, suggesting possible protective effects on hearing abilities. However, those results should be interpreted with caution and more (longitudinal) studies are needed to truly elucidate the association between specific food groups and hearing thresholds.

In our longitudinal analysis of body composition and age-related hearing loss, no significant associations were found, but effects estimates remained about the same as in the cross-sectional analysis. The absence of a significant effect might be explained by the fact that relative few people had a hearing assessment at follow-up but more likely, that the time interval might have been too short. To our knowledge, there are only two studies of a longitudinal origin [7] [21], in which the first does find a significant association and the latter does not. Clearly more evidence is needed to make any definite conclusions about body composition being a modifiable risk factor to prevent age-related hearing loss.

In conclusion, this study shows no association of diet quality with hearing loss and that a higher BMI is associated with hearing thresholds. This association with BMI is mainly driven by a higher FMI, suggesting involvement of metabolic and cardiovascular mechanisms, which may affect the cochlear function and suggesting that FMI is a better measure of body composition in age-related hearing loss. Whether a healthy body composition could serve as a preventive strategy for age-related hearing loss and thereby reducing the adverse consequences of hearing loss in older adults requires further longitudinal population-based research.

## MATERIALS AND METHODS

### Study design and subjects

This study was embedded in the Rotterdam Study, a population-based prospective cohort study in the Netherlands [30]. From 2011 onwards, hearing assessment was implemented in the study protocol, currently adding up to 6,494 audiograms. From this group, we excluded participants with no information on body composition (N = 1,155) and no information on diet quality (N = 868). Dietary assessment was performed between 2006 and 2012, and assessment of body composition was performed between 2009 and 2014. We finally excluded all hearing assessments performed later than 2014 (N = 1,565) resulting in a total sample of 2,906 participants for the cross-sectional analysis. Of this group, 636 participants had a second hearing assessment between 2014 and 2016.

The Rotterdam Study has been approved by the medical ethics committee of the Erasmus MC (registration number MEC 02.1015) and by the Dutch Ministry of Health, Welfare and Sport (Population Screening Act WBO, license number 1071272-159521-PG). All participants provided written informed consent to participate in the study and to have their information obtained from treating physicians.

### Body composition

Information on body weight and length was obtained by physical examination and BMI was calculated (kg/m^2^) [30]. A total body dual-energy X-ray absorptiometry (DXA) – scan was made from which bone mass, lean mass, and fat mass in kilograms was determined [30]. With the information obtained from the DXA-scan we calculated fat mass index (FMI, kg/m^2^), and fat-free mass index (FFMI, kg/m^2^). In this division, the sum of FMI and FFMI is BMI.

### Diet quality

Diet quality was assessed with a validated self-administered semi-quantitative food-frequency questionnaire (FFQ) consisting of 389 items. The FFQ was found to be an appropriate measurement tool for ranking people according to their food intake [31]. As described in detail elsewhere [31], we evaluated adherence (yes/no) to fourteen items (vegetables, fruit, (whole) grains, fats, nuts, legumes, dairy, fish, tea, red and processed meat, alcohol, sugar-containing beverages, and salt) of the Dutch dietary guidelines. An overall diet score ranging from 0-14 was calculated by adding up the scores for the fourteen food groups [31]. The Dutch Dietary Guidelines are based on internationally used dietary guidelines and on international literature about health effects of diet, as described in detail elsewhere [31].

### Hearing levels

Audiometric assessment was performed in a soundproof booth by one health care professional [30]. A computer-based audiometry system (Decos Technology Group, version 210.2.6 with AudioNigma interface) and TDH-39 headphones were used.

Pure tone audiometry was performed to determine hearing thresholds in decibel (dB) hearing level, measured according to the ISO-standard 8253-1 (International Organization for Standardization [ISO], 2010). For both ears, air conduction (frequencies 0.25, 0.50, 1, 2, 4, and 8 kilohertz (kHz)) was tested. Masking was performed according to the method of Hood (Hood, 1960). The best hearing ear was determined by taking the average threshold over all frequencies. When hearing thresholds for both ears were equal, we alternately chose the left or right ear. The low-frequency hearing threshold is the average of 0.25, 0.50, and 1 kHz and the high-frequency hearing threshold is the average of 2, 4, and 8 kHz. We excluded participants with an air-bone gap of 15 dB or more in the better ear to eliminate conductive hearing loss.

### Covariates

Information on smoking was collected through self-report and categorized into never, former and current [30]. Educational level was categorized as primary, lower, middle or higher [30]. Systolic and diastolic blood pressure was measured twice using a random zero-sphygmomanometer. Glucose was determined using the Hexokinase method. Using an automatic enzymatic procedure serum total cholesterol and high-density lipoprotein cholesterol were measured from fasting blood samples [30]. Hypertension was defined as systolic blood pressure ≥ 160 mmHg and/or diastolic blood pressure ≥ 90 mmHg and/or the use of blood pressure lowering medication [30]. Hypercholesterolemia was defined as total cholesterol concentration ≥ 6.2 mmol/L and/or the use of lipid-lowering medication [30]. Type 2 diabetes was defined as having fasting blood glucose concentration > 7.0 mmol/L and/or non-fasting blood glucose > 11.1 mmol/L and/or use of glucose-lowering medication. The LASA Physical Activity Questionnaire was used to assess physical activity. Physical activity data were recalculated into metabolic equivalent of task hours per week [32].

### Statistical analysis

The association of body composition (BMI, FMI, and FFMI) and of diet quality (overall score, and intake of the individual components per 10 grams) with hearing loss (all, low, and high frequencies) was examined using multivariable linear regression models. In the first model we adjusted for sex, age, age^2^, and education. In the second model we additionally adjusted for total brain volume, education, physical activity, smoking (current and past), energy intake, alcohol intake, hypertension, hypercholesterolemia and type 2 diabetes. In the third model for body composition, we additionally adjusted for diet quality score and in the second model for diet quality, we additionally adjusted for BMI. For hearing thresholds at follow-up, the same regression models were used, additionally corrected for hearing levels at baseline and for time between hearing assessments.

We explored whether associations differed by sex and if effects differed across BMI groups.

Missing data on covariates (<1%) were imputed using the multiple imputation algorithm (5 imputations) of SPSS. IBM SPSS statistics for Windows, version 24.0 (International Business Machines Corporation, Armonk, New York) was used.

### Data availability

Data can be obtained on request. Requests should be directed toward the management team of the Rotterdam Study (secretariat.epi@erasmusmc.nl), which has a protocol for approving data requests. Because of restrictions based on privacy regulations and informed consent of the participants, data cannot be made freely available in a public repository.

## SUPPLEMENTARY MATERIAL

Supplementary Tables S1-S6
