# Supplementary material for: The association between obesity, diet quality and hearing loss in older adults
Source: Aging (Albany NY). 2019 Jan 4;11(1):48–62. doi: 10.18632/aging.101717 (PMC6339793; doi:10.18632/aging.101717)
Supplement: Supplementary Tables S1-S6 [file aging-11-101717-s001.docx]

**Table S1. The longitudinal association between body composition and change in hearing thresholds.**

|  | All frequencies | Low frequencies | High frequencies |
| --- | --- | --- | --- |
|  | Difference (CI 95%) | Difference (CI 95%) | Difference (CI 95%) |
|  |  | **Model 1** |  |
| Body mass index (SD) | 0.22 (-0.16, 0.60) | 0.26 (-0.18, 0.69) | 0.15 (-0.37, 0.66) |
| Fat mass index (SD) | 0.11 (-0.30, 0.51) | 0.10 (-0.36, 0.56) | 0.02 (-0.53, 0.57) |
| Fat free mass index (SD) | 0.41 (-0.04, 0.85) | **0.52 (0.01, 1.03)** | 0.37 (-0.24, 0.98) |
|  |  | **Model 2** |  |
| Body mass index (SD) | 0.58 (-0.24, 1.41) | 0.37 (-0.64, 1.37) | 0.79 (-0.27, 1.85) |
| Fat mass index (SD) | 0.46 (-0.43, 1.36) | 0.12 (-0.96, 1.21) | 0.56 (-0.59, 1.71) |
| Fat free mass index (SD) | 0.71 (-0.22, 1.64) | 0.72 (-0.40, 1.84) | 1.08 (-0.11, 2.26) |
|  |  | **Model 3** |  |
| Body mass index (SD) | 0.58 (-0.25, 1.41) | 0.41 (-0.59, 1.42) | 0.79 (-0.27, 1.86) |
| Fat mass index (SD) | 0.42 (-0.47, 1.32) | 0.21 (-0.89, 1.30) | 0.57 (-0.59, 1.73) |
| Fat free mass index (SD) | 0.75 (-0.18, 1.68) | 0.70 (-0.42, 1.82) | 1.08 (-0.12, 2.27) |

All frequencies (0.25, 0.50, 1, 2, 4, and 8 kHz); low frequencies (0.25, 0.50, and 1 kHz); high frequencies (2, 4, and 8 kHz). Difference; represents the difference in dB per one SD higher body mass index, fat mass index, fat free mass index, and lean mass index. CI: confidence interval. Model 1: adjusted for sex, age, age^2^, education, hearing loss at baseline, and age difference between hearing assessments. Model 2: additionally adjusted for brain volume, energy intake, physical activity, smoking, alcohol, hypertension, hypercholesterolemia and type 2 diabetes. Model 3: additionally adjusted for diet quality score. Significant effect estimates (p<0.05) are indicated in **bold**.

**Table S2. The cross-sectional association between diet quality, food groups and hearing thresholds – model 1.**

|  | All frequencies | Low frequencies | High frequencies |
| --- | --- | --- | --- |
|  | Difference (CI 95%) | Difference (CI 95%) | Difference (CI 95%) |
| Diet quality | -0.15 (-0.34, 0.05) | **-0.21 (-0.40, -0.02)** | -0.06 (-0.34, 0.21) |
| Vegetables | -0.01 (-0.03, 0.01) | **-0.02 (-0.03, -0.00)** | -0.00 (-0.03, 0.03) |
| Fruit | -0.01 (-0.02, 0.00) | -0.01 (-0.02, 0.00) | -0.01 (-0.03, 0.00) |
| Whole grain products | -0.01 (-0.06, 0.04) | -0.04 (-0.07, 0.00) | 0.02 (-0.06, 0.09) |
| Whole grains/total grains ratio | -0.01 (-0.05, 0.03) | -0.02 (-0.05, 0.02) | -0.00 (-0.06, 0.05) |
| Legumes | 5.40 (-2.55, 13.35) | 5.05 (-1.61, 11.71) | 4.62 (-6.75, 15.99) |
| Nuts | -0.02 (-0.25, 0.21) | -0.06 (-0.25, 0.13) | 0.03 (-0.30, 0.35) |
| Dairy | 0.00 (-0.01, 0.02) | -0.00 (-0.02, 0.01) | 0.01 (-0.01, 0.03) |
| Fish | -0.07 (-0.24, 0.09) | -0.11 (-0.24, 0.03) | -0.07 (-0.30, 0.16) |
| Tea | **-0.02 (-0.03, -0.00)** | -0.01 (-0.03, 0.00) | -0.02 (-0.05, 0.00) |
| Unsaturated fats/total fats ratio | 0.04 (-0.07, 0.16) | 0.05 (-0.05, 0.15) | 0.05 (-0.12, 0.21) |
| Salt | 0.00 (-0.00, 0.00) | -0.00 (-0.00, 0.00) | 0.00 (-0.00, 0.00 |
| Alcohol | -0.06 (-0.30, 0.19) | -0.06 (-0.26, 0.15) | -0.05 (-0.40, 0.29) |
| Red and processed meat | 0.05 (-0.02, 0.13) | **0.06 (0.00, 0.13)** | 0.04 (-0.07, 0.14) |
| Sugar containing beverages | -0.03 (-0.06, 0.01) | -0.02 (-0.04, 0.01) | -0.0.3 (-0.07, 0.01) |

All frequencies (0.25, 0.50, 1, 2, 4, and 8 kHz); low frequencies (0.25, 0.50, and 1 kHz); high frequencies (2, 4, and 8 kHz). Difference represents difference in dB per 1 point increase in diet quality score on a scale ranging from 0 to 14 or a 10 gram increase for the individual food components. CI: confidence interval. Adjusted for sex, age, age^2^, and education (model 1). Significant effect estimates (p<0.05) are indicated in **bold**.

**Table S3. The cross-sectional association between diet quality, food groups and hearing thresholds – model 2.**

|  | All frequencies | Low frequencies | High frequencies |
| --- | --- | --- | --- |
|  | Difference (CI 95%) p-value | Difference (CI 95%) p-value | Difference (CI 95%) p-value |
| Diet quality | -0.11 (-0.35, 0.14) | -0.17 (-0.38, 0.04) | -0.06 (-0.42, 0.29) |
| Vegetables | -0.01 (-0.04, 0.01) | -0.02 (-0.04, 0.01) | -0.01 (-0.05, 0.03) |
| Fruit | -0.01 (-0.02, 0.01) | -0.00 (-0.02, 0.01) | -0.01 (-0.03, 0.01) |
| Whole grain products | -0.00 (-0.06, 0.06) | -0.03 (-0.08, 0.02) | 0.03 (-0.06, 0.12) |
| Whole grains/total grains ratio | 0.00 (-0.05, 0.06) | -0.00 (-0.05, 0.05) | 0.01 (-0.07, 0.10) |
| Legumes | 6.91 (-2.75, 16.57) | 8.03 (-0.02, 16.07) | 4.49 (-9.49, 18.47) |
| Nuts | -0.03 (-0.31, 0.25) | -0.03 (-0.27, 0.20) | -0.02 (-0.23, 0.19) |
| Dairy | 0.00 (-0.01, 0.02) | -0.01 (-0.02, 0.01) | 0.02 (-0.01, 0.04) |
| Fish | -0.05 (-0.25, 0.15) | -0.09 (-0.25, 0.08) | -0.04 (-0.33, 0.25) |
| Tea | -0.01 (-0.03, 0.01) | -0.01 (-0.02, 0.01) | -0.02 (-0.05, 0.01) |
| Unsaturated fats/total fats ratio | 0.14 (-0.02, 0.29) | **0.16 (0.03, 0.29)** | 0.14 (-0.09, 0.36) |
| Salt | -0.00 (-0.00, 0.00) | -0.00 (-0.00, 0.00) | 0.00 (-0.00, 0.01) |
| Alcohol | -0.07 (-0.37, 0.22) | -0.06 (-0.31, 0.18) | -0.10 (-0.52, 0.33) |
| Red and processed meat | 0.05 (-0.04, 0.14) | 0.06 (-0.02, 0.15) | 0.02 (-0.05, 0.09) |
| Sugar containing beverages | -0.04 (-0.08, -0.00) | **-0.04 (-0.07, -0.01)** | -0.04 (-0.09, 0.01) |

All frequencies (0.25, 0.50, 1, 2, 4, and 8 kHz); low frequencies (0.25, 0.50, and 1 kHz); high frequencies (2, 4, and 8 kHz). Difference represents difference in dB per 1 point increase in diet quality score on a scale ranging from 0 to 14 or a 10 gram increase for the individual food components. CI: confidence interval. Adjusted for sex, age, age^2^, education, physical activity, smoking (former and current), alcohol intake, hypertension, hypercholesterolemia, prevalent diabetes mellitus, total brain volume and energy intake (model 2). We did not adjust for alcohol intake in grams in the assessment of alcohol with hearing thresholds. Significant effect estimates (p<0.05) are indicated in **bold.**

**Table S4. The longitudinal association between diet quality, food groups and change in hearing thresholds – model 1.**

|  | All frequencies | Low frequencies | High frequencies |
| --- | --- | --- | --- |
|  | Difference (CI 95%) | Difference (CI 95%) | Difference (CI 95%) |
| Diet quality | -0.02 (-0.20, 0.17) | -0.00 (-0.21, 0.20) | -0.08 (-0.34, 0.18) |
| Vegetables | -0.01 (-0.03, 0.01) | 0.00 (-0.02, 0.03) | -0.02 (-0.05, 0.01) |
| Fruit | 0.00 (-0.01, 0.01) | 0.00 (-0.01, 0.01) | 0.01 (-0.01, 0.02) |
| Whole grain products | -0.02 (-0.07, 0.03) | -0.04 (-0.09, 0.02) | -0.01 (-0.08, 0.06) |
| Whole grains/total grains ratio | -0.01 (-0.05, 0.03) | -0.01 (-0.06, 0.04) | -0.01 (-0.06, 0.05) |
| Legumes | 2.67 (-5.11, 10.45) | 4.41 (-4.34, 13.15) | 0.36 (-10.45, 11.17) |
| Nuts | -0.00 (-0.26, 0.25) | 0.17 (-0.12, 0.45) | -0.20 (-0.55, 0.16) |
| Dairy | 0.01 (-0.01, 0.02) | 0.00 (-0.02, 0.02) | 0.01 (-0.02, 0.03) |
| Fish | 0.11 (-0.05, 0.27) | **0.24 (0.06, 0.42)** | 0.02 (-0.21, 0.24) |
| Tea | -0.01 (-0.03, 0.01) | -0.01 (-0.03, 0.01) | -0.01 (-0.04, 0.01) |
| Unsaturated fats/total fats ratio | -0.07 (-0.20, 0.07) | -0.11 (-0.26, 0.05) | -0.03 (-0.22, 0.16) |
| Salt | 0.00 (-0.00, 0.00) | 0.00 (-0.00, 0.00) | 0.00 (-0.00, 0.00) |
| Alcohol | 0.02 (-0.23, 0.26) | 0.16 (-0.12, 0.44) | -0.13 (-0.48, 0.21) |
| Red and processed meat | 0.05 (-0.02, 0.13) | **0.09 (0.00, 0.17)** | 0.03 (-0.07, 0.14) |
| Sugar containing beverages | 0.03 (-0.01, 0.06) | 0.02 (-0.02, 0.06) | 0.00 (-0.05, 0.05) |

All frequencies (0.25, 0.50, 1, 2, 4, and 8 kHz); low frequencies (0.25, 0.50, and 1 kHz); high frequencies (2, 4, and 8 kHz). Difference represents difference in dB per 1 point increase in diet quality score on a scale ranging from 0 to 14 or a 10 gram increase for the individual food components. CI: confidence interval. Adjusted for sex, age at baseline, age^2^, education, time between baseline and follow-up hearing assessment and baseline hearing thresholds (model 1). Significant effect estimates (p<0.05) are indicated in **bold**.

**Table S5. The longitudinal association between diet quality, food groups and change in hearing thresholds – model 2.**

|  | All frequencies | Low frequencies | High frequencies |
| --- | --- | --- | --- |
|  | Difference (CI 95%) | Difference (CI 95%) | Difference (CI 95%) |
| Diet quality | -0.12 (-0.53, 0.30) | -0.19 (-0.73, 0.35) | -0.20 (-0.78, 0.39) |
| Vegetables | -0.04 (-0.08, 0.00) | -0.02 (-0.08, 0.03) | **-0.06 (-0.12, -0.01)** |
| Fruit | 0.01 (-0.01, 0.04) | -0.00 (-0.03, 0.03) | 0.03 (-0.00, 0.06) |
| Whole grain products | 0.05 (-0.06, 0.16) | 0.03 (-0.11, 0.16) | 0.05 (-0.10, 0.20) |
| Whole grains/total grains ratio | 0.03 (-0.08, 0.13) | 0.04 (-0.09, 0.16) | 0.03 (-0.11, 0.16) |
| Legumes | 0.99 (-14.55, 16.53) | 4.30 (-14.33, 22.93) | -6.66 (-26.96, 13.65) |
| Nuts | **-0.97 (-1.55, -0.40)** | -0.67 (-1.38, 0.04) | **-1.14 (-1.91, -0.37)** |
| Dairy | 0.02 (-0.02, 0.06) | 0.00 (-0.05, 0.05) | 0.03 (-0.03, 0.08) |
| Fish | 0.18 (-0.14, 0.50) | 0.21 (-0.18, 0.60) | -0.03 (-0.45, 0.39) |
| Tea | -0.01 (-0.05, 0.03) | -0.02 (-0.06, 0.03) | 0.00 (-0.05, 0.05) |
| Unsaturated fats/total fats ratio | -0.02 (-0.36, 0.32) | -0.03 (-0.45, 0.38) | 0.15 (-0.30, 0.60) |
| Salt | 0.00 (-0.01, 0.01) | 0.00 (-0.00, 0.01) | -0.00 (-0.01, 0.01) |
| Alcohol | -0.10 (-0.71, 0.51) | -0.37 (-1.10, 0.37) | 0.16 (-0.65, 0.97) |
| Red and processed meat | 0.03 (-0.16, 0.22) | 0.16 (-0.07, 0.39) | -0.02 (-0.27, 0.24) |
| Sugar containing beverages | 0.03 (-0.05, 0.10) | 0.06 (-0.04, 0.15) | -0.07 (-0.17, 0.04) |

All frequencies (0.25, 0.50, 1, 2, 4, and 8 kHz); low frequencies (0.25, 0.50, and 1 kHz); high frequencies (2, 4, and 8 kHz). Difference represents difference in dB per 1 point increase in diet quality score on a scale ranging from 0 to 14 or a 10 gram increase for the individual food components. CI: confidence interval. Adjusted for sex, age at baseline, age^2^, education, time between baseline and follow-up hearing assessment, baseline hearing thresholds, total brain volume, energy intake, physical activity, smoking (former and current), alcohol intake, hypertension, hypercholesterolemia and prevalent diabetes mellitus (model 2). We did not adjust for alcohol intake in grams in the assessment of alcohol with hearing thresholds. Significant effect estimates (p<0.05) are indicated in **bold**.

**Table S6. The longitudinal association between diet quality, food groups and change in hearing thresholds – model 3.**

|  | All frequencies | Low frequencies | High frequencies |
| --- | --- | --- | --- |
|  | Difference (CI 95%) | Difference (CI 95%) | Difference (CI 95%) |
| Diet quality | -0.19 (-0.63, 0.26) | -0.21 (-0.75, 0.33) | -0.22 (-0.80, 0.36) |
| Vegetables | **-0.05 (-0.09, -0.00)** | -0.03 (-0.08, 0.03) | -0.07 (-0.13, -0.01) |
| Fruit | 0.01 (-0.01, 0.04) | -0.00 (-0.03, 0.03) | 0.03 (-0.00, 0.06) |
| Whole grain products | 0.05 (-0.07, 0.16) | 0.02 (-0.11, 0.16) | 0.05 (-0.10, 0.19) |
| Whole grains/total grains ratio | 0.02 (-0.09, 0.12) | 0.03 (-0.10, 0.16) | 0.01 (-0.12, 0.15) |
| Legumes | 0.01 (-15.55, 15.57) | 3.67 (-15.05, 22.40) | -7.82 (-28.09, 12.45) |
| Nuts | **-0.95 (-1.52, -0.37)** | -0.65 (-1.36, 0.07) | **-1.10 (-1.87, -0.33)** |
| Dairy | 0.02 (-0.02, 0.06) | 0.00 (-0.05, 0.05) | 0.03 (-0.03, 0.08) |
| Fish | 0.13 (-0.20, 0.46) | 0.18 (-0.22, 0.58) | -0.11 (-0.54, 0.32) |
| Tea | -0.01 (-0.05, 0.03) | -0.01 (-0.06, 0.03) | 0.01 (-0.04, 0.06) |
| Unsaturated fats/total fats ratio | -0.02 (-0.36, 0.32) | -0.03 (-0.45, 0.38) | 0.15 (-0.30, 0.60) |
| Salt | 0.00 (-0.01, 0.01) | 0.00 (-0.00, 0.01) | -0.00 (-0.01, 0.01) |
| Alcohol | -0.05 (-0.67, 0.56) | -0.33 (-1.07, 0.40) | 0.22 (-0.59, 1.03) |
| Red and processed meat | 0.03 (-0.16, 0.22) | 0.16 (-0.07, 0.39) | -0.01 (-0.26, 0.24) |
| Sugar containing beverages | 0.03 (-0.05, 0.10) | 0.06 (-0.04, 0.15) | -0.06 (-0.17, 0.04) |

All frequencies (0.25, 0.50, 1, 2, 4, and 8 kHz); low frequencies (0.25, 0.50, and 1 kHz); high frequencies (2, 4, and 8 kHz). Difference represents difference in dB per 1 point increase in diet quality score on a scale ranging from 0 to 14 or a 10 gram increase for the individual food components. CI: confidence interval. Adjusted for sex, age at baseline, age^2^, education, time between baseline and follow-up hearing assessment, baseline hearing thresholds, total brain volume, energy intake, physical activity, smoking (former and current), alcohol intake, hypertension, hypercholesterolemia, prevalent diabetes mellitus and BMI (model 3). We did not adjust for alcohol intake in grams in the assessment of alcohol with hearing thresholds. Significant effect estimates (p<0.05) are indicated in **bold**.
